# Allograft Cellular Bone Matrix in Extreme Lateral Interbody Fusion: Preliminary Radiographic and Clinical Outcomes

**DOI:** 10.1100/2012/263637

**Published:** 2012-12-02

**Authors:** Antoine G. Tohmeh, Blake Watson, Mirna Tohmeh, Xavier J. Zielinski

**Affiliations:** ^1^Northwest Orthopaedic Specialists, Spokane, WA 99208, USA; ^2^Inland Imaging, Spokane, WA 99204, USA; ^3^Spine Research Institute of Spokane, PLLC, USA

## Abstract

*Introduction*. Extreme lateral interbody fusion (XLIF) is a minimally disruptive alternative for anterior lumbar interbody fusion. Recently, synthetic and allograft materials have been increasingly used to eliminate donor-site pain and complications secondary to autogenous bone graft harvesting. The clinical use of allograft cellular bone graft has potential advantages over autograft by eliminating the need to harvest autograft while mimicking autograft's biologic function. The objective of this study was to examine 12-month radiographic and clinical outcomes in patients who underwent XLIF with Osteocel Plus, one such allograft cellular bone matrix. *Methods*. Forty (40) patients were treated at 61 levels with XLIF and Osteocel Plus and included in the analysis. *Results*. No complications were observed. From preoperative to 12-month postoperative followup, ODI improved 41%, LBP improved 55%, leg pain improved 43.3%, and QOL (SF-36) improved 56%. At 12 months, 92% reported being “very” or “somewhat” satisfied with their outcome and 86% being either “very” or “somewhat likely” to choose to undergo the procedure again. Complete fusion was observed in 90.2% (55/61) of XLIF levels. *Conclusions*. Complete interbody fusion with Osteocel Plus was shown in 90.2% of XLIF levels, with the remaining 9.8% being partially consolidated and progressing towards fusion at 12 months.

## 1. Introduction 

Conventional surgical approaches for lumbar interbody fusion using open exposures have been associated with high rates of approach-related morbidity and extensive soft-tissue dissection [1–12]. Common complications include vascular, visceral, and reproductive complications after conventional direct anterior lumbar interbody fusion (ALIF) [1, 8–12] and nerve injury following posterior/transforaminal lumbar interbody fusion (P/TLIF) [11, 13–16]. Minimally invasive (MIS) approaches for lumbar interbody fusion have gained in popularity over the past decade as nonendoscopic MIS approaches have been developed to allow for greater reproducibility without extended learning curves [17–21]. 

These modern MIS approaches include mini-open direct anterior ALIF [17, 21], MIS TLIF [16, 22, 23], and mini-open lateral ALIF (extreme lateral interbody fusion (XLIF)) [19]. The miniopen lateral ALIF approach accesses the lumbar spine through a mini-open, 90° off-midline, retroperitoneal, transpsoas approach and largely avoids vascular, visceral, reproductive, and neural complications common to conventional direct anterior ALIF and P/TLIF approaches, with minimal incidence of infection, transfusion, and markedly shorter hospitalization when compared with traditional open approaches [24, 25]. The primary potential risk when performing the XLIF procedure is injury or irritation to nerves in and around the iliopsoas muscle. However, the risk to motor nerves is mitigated through the use of advanced neurophysiologic monitoring, which provides real-time, surgeon-directed, discrete threshold electromyographic responses in directional orientations, to supply geographic information on the position and distance of motor nerves relative to instrumentation [26–29]. As a result, the most commonly reported complications following the XLIF procedure have been mild hip/thigh numbness and hip-flexion weakness, which typically resolve within 6 weeks following surgery [24, 26, 30–32]. 

The XLIF procedure preserves the anterior and posterior longitudinal ligaments and the exposure allows for broad disc space preparation and placement of a wide intervertebral cage that spans the lateral borders of the ring apophysis, resting on cortical bone to resist implant subsidence. The large aperture of the interbody cage allows for the placement of bone graft material and a large surface area for fusion.

In addition to minimizing approach-related morbidity through the use of mini-open approaches, synthetic and allograft materials have been increasingly used to decrease donor-site pain and complications secondary to autogenous bone graft harvesting [33, 34]. One such example is allograft cellular bone matrix (ACBM), which retains its native bone-forming cells, including mesenchymal stem cells and osteoprogenitor cells. Like autograft, ACBM provides all three physiologic mechanisms that are instrumental in normal bone healing, including osteoconduction, osteoinduction, and osteogenesis [35, 36]. 

The clinical use of allograft cellular bone graft has potential advantages over autograft in spinal fusion procedures by eliminating the need to harvest autogenous bone graft—which can lead to infection, increased blood loss and operative time, chronic donor-site pain, and iliac crest fracture, in addition to the fact that the immediate postoperative pain associated with bone harvesting defeats the purpose of minimally invasive surgery [33, 34]—while still providing all three elements necessary for bone healing. Of note, the mesenchymal stem cells (MSCs) contained in ACBM, which have multipotential differentiation abilities in vivo, can differentiate into mesenchymal tissues, namely, bone and cartilage through osteogenesis and chondrogenesis, respectively.

The object of this study was to examine 12-month radiographic and clinical outcomes in patients who underwent extreme lateral interbody fusion with ACBM as the sole bone growth substrate.

## 2. Methods 

**Figure 1:**
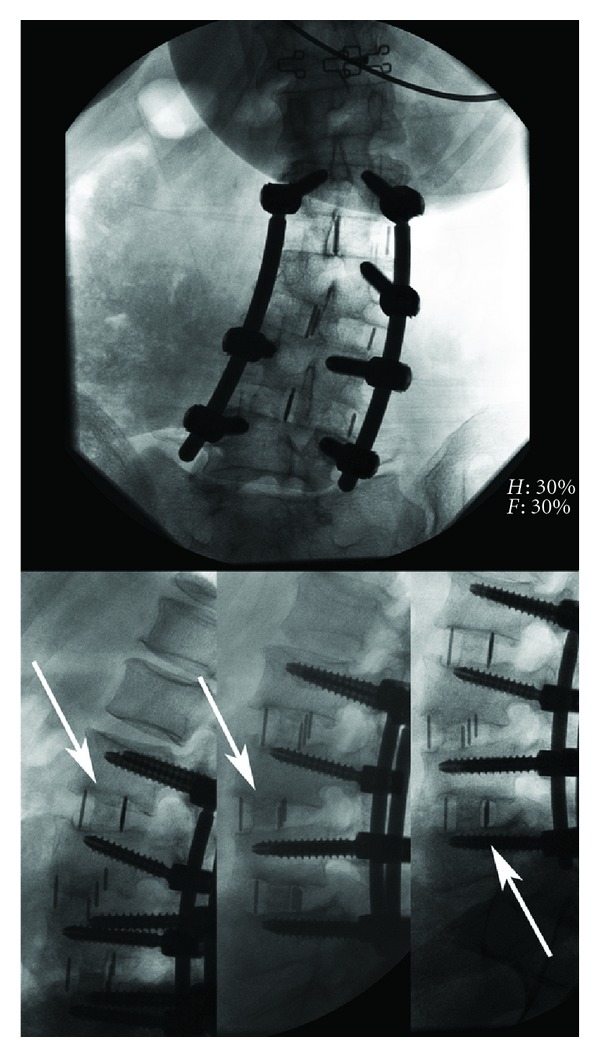
Anterior and lateral fluoroscopy-guided, level-by-level radiography. Note that parallel endplates were obtained on lateral radiography using this technique (white arrows), avoiding X-ray parallax.

Clinical and radiographic data were collected through a prospective registry examining patient outcomes after undergoing XLIF (NuVasive, Inc. San Diego, CA, USA) for lumbar interbody fusion using Osteocel Plus (NuVasive, Inc.) ACBM at a single institution in Spokane, WA, USA, from February 2009 to February 2011. Neurophysiologic monitoring was performed in all cases using NV JJB/M5 (NuVasive, Inc.). Inclusion criteria in the current post hoc analysis included having been treated with XLIF at any level with ACBM as the sole bone graft material, having had at least 12-month followup with fluoroscopy-guided, level-by-level, radiography (FGX) (to eliminate parallax and make accurate PER-level assessments, Figure 1) or computed tomography (CT) assessed by a third-party reviewer to determine extent of bony fusion in XLIF levels. Fusion criteria used are as follows.

**(i) Complete Fusion**

Complete ossification with some component of endplate involvement.

**(ii) Incomplete/Progressing Fusion**

Ossification in cage that abuts one or both endplates without evidence of endplate involvement or continuous bridging bone.

**(iii) Indeterminate**

Clear lucencies at endplates with or without ossification in the cage.

In total, forty (40) patients met all inclusion criteria and were included in these analyses. Thirty-nine (98%) patients had fusion status evaluated using FGX, while one (3%) was assessed by CT. Of the 40 patients assessed for fusion, 35 (88%) had both preoperative and 12-month postoperative functional outcome scores, which were evaluated as a subset of the sample. 

**Figure 2:**
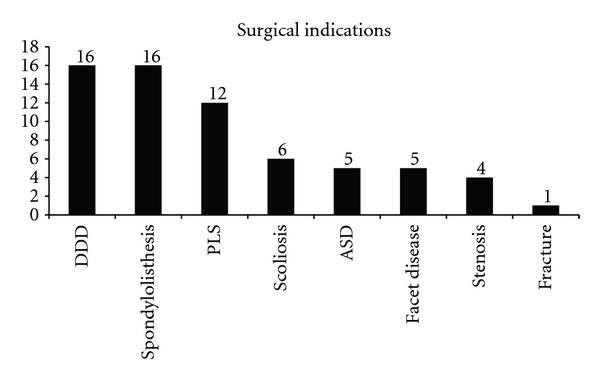
Graph showing the number of patients with each indication.

Mean age of the patients was 60 years (range 36–82 years) with a mean body mass index of 28.1 (range 20–38). Comorbidities were common, with 48% of patients having a history of heart disease, 20% with diabetes mellitus, 13% were smokers, and 65% had undergone prior lumbar spine surgery. Of those with prior spine surgery, a total of 20 surgeries had been performed: one (5%) discectomy, 7 (25%) instrumented fusions, and 18 (70%) laminectomies. Sixty-five (65) indications for treatment were noted in the 40 patients (mean 1.6 per patient). The most common diagnoses (multiple per patient) were degenerative disc disease (DDD) (40%), spondylolisthesis (40%), and postlaminectomy syndrome (30%). A listing of all diagnoses can be found in Figure 2 and full baseline characteristics are included in Table 1. 

**Figure 3:**
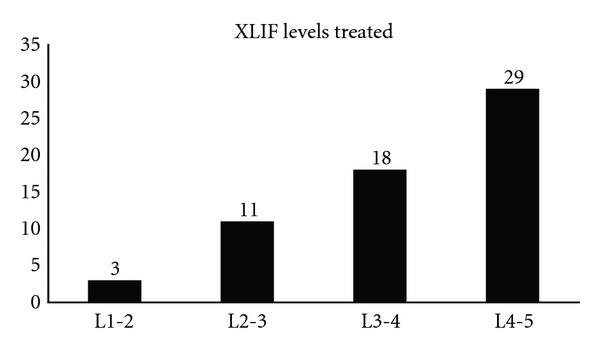
Graph showing the number of each level treated with extreme lateral interbody fusion.

In total, 68 levels were treated in 40 patients (mean 1.7 levels per patient, range 1–3) between L1 and S1. Of these, 61 levels from L1 to L5 were treated with XLIF and 7 levels at L5–S1 underwent TLIF. The most common levels treated were L4-5 (48%) and L3-4 (30%). A complete listing of XLIF levels treated is included in Figure 3. Supplemental internal fixation was used in all patients and included lateral plating in 38% and bilateral transpedicular fixation in 63% of patients. A direct decompression was performed in 13 (33%) patients. In all cases the sole biologic material used was Osteocel Plus. Complete treatment characteristics are included in Table 2. 

For a historical control, a review of the literature was performed to examine fusion and pseudoarthrosis revision rates in lumbar spinal fusion using autograft as the bone graft material. A total of 24 peer-reviewed articles [8, 13, 15, 18, 37–56] following 2,026 patients treated with lumbar fusion using autograft were reviewed and analyzed. Articles reviewed were determined to have the following levels of evidence: [57] I : 7 (27%), II : 3 (14%), III : 2 (9%), and IV : 11 (50%) and procedures examined included conventional direct anterior ALIF in 11 (50%) studies, TLIF in 2 (9%), PLIF in 5 (23%), and posterolateral fusion in 5 (23%). 

## 3. Results 

Mean initial patient positioning from induction of anesthesia to initial incision was 32 minutes, mean interbody procedure time (all levels) was 57 minutes, mean repositioning for posterior procedure time (where applicable) was 27 minutes, and mean posterior/fixation procedure time was 73 minutes. Mean total positioning time was 59 minutes (range 25–109 mins), mean total operative time was 122 minutes (range 49–274 mins), and mean total time in the operative field was 178 minutes (range 75–342 mins). Mean XLIF blood loss was 54 cc per patient, with mean 129 cc for combined anterior and posterior procedures (Table 2). No complications were observed. One patient underwent reoperation at an adjacent level with XLIF for DDD. A second patient has developed new symptoms in an L5–S1 distribution, with a potential pseudoarthrosis at an L5–S1 TLIF, though symptom etiology and course of treatment are currently unknown.

Thirty-five (35) patients also had pre- and 12-month postoperative functional outcome scores. From preoperative to 12-month postoperative followup, Oswestry disability index (ODI) improved 41% (45.7 to 27.1), low back pain (LBP) (visual analog scale (VAS)) improved 55% (7.4 to 3.4), leg pain (LP) improved 43% (6.8 to 3.8), and quality of life (SF-36) improved 55.6% (41.8 to 65.0). All clinical variables were statistically significantly different from pre- to postoperative time points, *P* < 0.05. Of the 36 (90%) of patients who completed a satisfaction questionnaire at 12-month postoperatively, 92% reported being either “very” or “somewhat” satisfied with their outcome and 86% reported being either “very” or “somewhat likely” to have undergone the same procedure had their outcome been known in advance.

**Figure 4:**
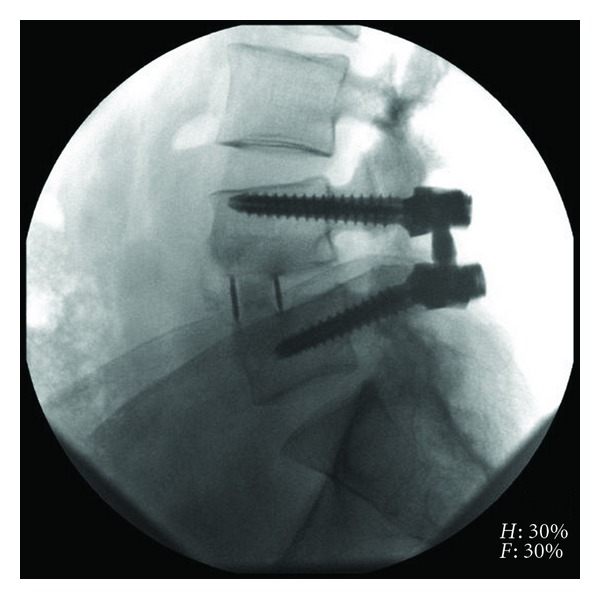
Lateral radiograph of L4-5 extreme lateral interbody fusion with Osteocel Plus at 12-month postoperative.

**Figure 5:**
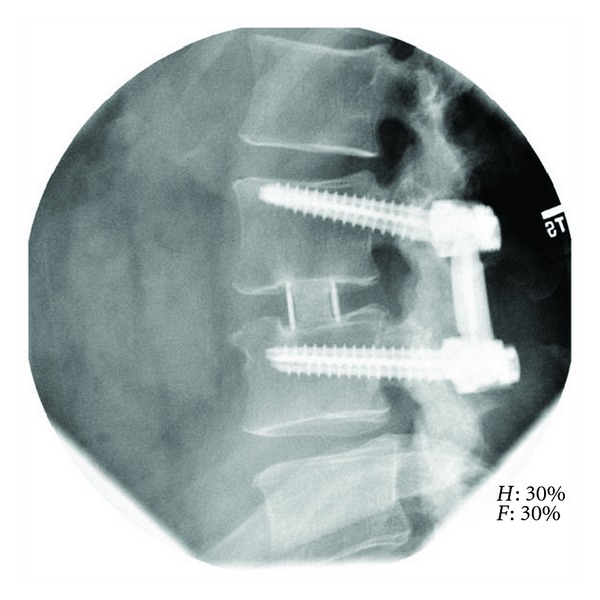
Lateral radiograph of L3-4 extreme lateral interbody fusion with Osteocel Plus at 12-month postoperatively.

Complete fusion was observed in 90.2% (55/61) of XLIF levels. Five (5) levels were assessed as incompletely fused, where ossification was present in the cage, but complete trabecular bridging had not yet occurred. In these instances, 3 of 6 were in multilevel cases (two 3-level, one 2-level case). One level was assessed as indeterminately fused or showing clear lucencies at endplates with or without ossification in the cage. Examples of completely fused XLIF levels can be found in Figures 4 and 5. Any evidence (≥1 mm) of radiographic subsidence was observed in 20 of 61 levels (32.8%) (Figure 4), though symptoms did not correlate with subsidence and no revisions occurred. 

### 3.1. Literature Review

In the review of 24 studies and 2,026 patients undergoing lumbar fusion using autograft, the weighted average for fusion rate was 87.6% (range 44%–100%) and the pseudoarthrosis revision rate was 3.8% (range 0%–28.1%) [8, 13, 15, 18, 37–51, 53–56]. 

## 4. Discussion

Evidence of bony fusion following lumbar fusion procedures is an important measure to gauge the long-term integrity of the fused segment and, as such, is generally valued alongside clinical outcomes as a primary surgical outcome. Historically, the “gold standard” biologic graft material to assist in the fusion process in spine fusion has been autograft, due to its inherent biologic properties of osteoconduction, osteoinduction, and osteogenesis, which are all required to form bone [35, 36]. However, the morbidity associated with autologous bone graft harvest from the iliac crest can be substantial and long lasting. In a review by Dimitriou et al. [33] of 81 studies of iliac crest bone graft (ICBG) harvesting in 6,449 patients, the authors found an ICBG harvest-related morbidity rate of 19.4%, the primary complications being chronic donor-site pain (7.8%), persistent dysesthesias at the harvest site (4.8%), hematomas/seromas (2.1%), and infection (1.4%). Similarly, Kim et al. prospectively followed 104 patients for one year to examine long-term pain and complications related to iliac crest harvesting for autologous bone graft in lumbar fusions [34]. At 12 months, the authors found that 16.5% of patients reported continued harvest-site pain and 29.1% reported persistent numbness. With respect to physical ability, the following activities were listed as difficult to perform due to continued harvest-site pain: 15.1% walking, 5.2% employment, 12.9% recreation, 14.1% household chores, 7.6% sexual activity, and 5.9% experienced harvest-site irritation when in contact with clothing [34]. 

Despite the associated morbidity, the precedent of ICBG as the standard bone graft material resulted, naturally, from there being limited allograft or synthetic alternatives until recently [35, 36]. And with allograft or synthetic materials, few inherently have all of the biological components required for fusion (and those few are only recently available, in the form of ACBMs), requiring combinations of materials to complete the biologic environment for the fusion cascade. One such example is osteoinductive graft material, such as bone morphogenic protein (rhBMP-2, Medtronic Sofamor Danek, Memphis, TN, USA), with osteoconductive ceramic synthetics and osteogenic bone marrow aspirate. Unfortunately, the use of rhBMP-2 in the spine has been shown in some studies to have a complication profile similar or greater than ICBG harvesting, though generally with high fusion rates. In a review of 31 articles discussing complications following BMP use in spine surgery, Mroz et al. [58] found a 44% mean rate of resorption/osteolysis, 25% rate of graft subsidence, 8% rate of ectopic bone growth, 27% rate of cage migration, 29% incidence of new onset radiculitis, and an inflammatory response to the collagen carrier in 29% of patients [58, 59]. 

As part of this study an extensive literature review was undertaken, for historical control, examining fusion and reoperation rates for pseudoarthroses in the autograft in spine fusion literature. In total, 24 peer-reviewed articles were included covering 2,026 patients. Overall fusion rate, using weighted average, was 87.6% with a range of reported fusion rates from 44% to 100%. Fusion rate in these studies was generally assessed at 24-month postoperatively or greater. Surgically revised pseudoarthroses occurred in a weighted average of 3.8% percent of cases with a range of 0%–7%. In one representative study from the review, McAfee et al. [52] and Blumenthal et al. [38] described 99 patients treated with the BAK-threaded cage through conventional direct anterior ALIF as the control group of the Charité randomized control FDA trial. In these patients with 2-year followup, the authors observed a fusion rate of 90.9% and pseudoarthrosis revision rate of 9.1%. Mean ORT was 114 minutes, blood loss was 209 cc, with an overall complication rate of 46.5% (minor and major), an 8% infection rate, 5.5% rate of retrograde ejaculation, and 18.2% rate of donor-site pain from ICBG harvest with a 30.5% improvement in ODI.

With respect to the current findings of MIS lateral ALIF with ACBM at 12-month followup, we observed a 90.2% solid fusion rate in 55/61 XLIF levels and 9.8% rate of incomplete or ongoing fusion in the remaining 4 (9.8%) levels. These data represent, to our knowledge, the first reported fusion results of ACBM in the human spine. There were no complications in this series, though there was a reoperation in one (3.7%) patient at the level above the index fusion for adjacent segment disease. Clinical outcomes, evaluated in a subset of patients, at 12 months showed an improvement on disability (ODI) of 41% and LBP and LP improved 55% and 43%, respectively, with a 56% increase in quality of life. In 92% of patients their general surgical outcome was rated as excellent or good and 86% would have undergone the same procedure again had their outcome been known in advance. With respect to fusion rate, the current results are higher than the weighted average from the historical control, and in-line with the high-end of the range of values of autograft in spine fusion, despite the fact that the literature generally reported 24-month or greater fusion rate compared to the 12-month followup of the current study. The high variability in the historical control is likely due to the lack of evaluation control and the retrospective nature of some of the literature, which is mitigated in part both by the relatively large number of publications and the resultant large patient series included. 

Limitations of the current study include the lack of a parallel control group with autograft or other bone graft material to directly compare fusion performance at the same institution. Additionally, final fusion is more typically assessed at 24-month postoperatively, which may account for our progressing fusion rate in 9.8% of patients. However, since this was designed as an early fusion assessment in a novel biologic graft material for fusion, we have limited claims in the conclusion to better reflect the preliminary nature of the results.

## 5. Conclusion

As of this writing, this work represents the first report of fusion and clinical results of allograft cellular bone matrix in the human spinal fusion procedures. These results, however, are preliminary, being only 12-month postoperatively in a relatively small number of patients. No complications were observed in this series of patients treated with XLIF for degenerative conditions of the lumbar spine. Functional outcomes, from a subset of the current series, improved significantly in pain, disability, and quality-of-life measurement from preoperative to 12-month postoperatively. The vast majority of patients reported being both satisfied with their outcome and would have chosen to undergo the procedure again had their outcome been known in advance. Complete interbody fusion, facilitated by allograft cellular bone matrix, was shown in 90.2% of XLIF levels, with 8.2% being partially consolidated. One level was of an indeterminate status. None of the index fused levels were revised. Subsequent work examining larger case series and comparisons with other biologic materials are needed to supplement these early favorable results.

## Figures and Tables

**Table 1:** Demographic information.

Characteristic	Statistic *n* = 40
Mean age in years (stdev) (range)	60.4 (12.2) (36–84)
Female (%)	20 (50)
Mean body mass index (BMI), (stdev) (range)	28.1 (4.4) (20–38)
Comorbidities (mean number per patient)	63 (1.6)
Comorbidity type	
Tobacco use (%)	5 (12.5)
Coronary artery disease (%)	19 (47.5)
Diabetes (%)	8 (20)
Chronic obstructive pulmonary disease (COPD) (%)	2 (5)
Steroid use (%)	3 (8)
Any prior lumbar/thoracic spine surgery (%)	26 (65)
Prior surgery type	*n* = 26
Discectomy (%)	1 (5)
Laminectomy (%)	18 (70)
Fusion (%)	7 (25)
Diagnoses (mean number per patient)	65 (1.6)
Degenerative disc disease (%)	16 (40)
Spondylolisthesis (%)	15 (38)
Postlaminectomy syndrome (%)	12 (30)
Adjacent segment disease (%)	5 (13)
Scoliosis (%)	6 (15)
Retrolisthesis (%)	1 (3)
Facet disease (%)	5 (13)
Stenosis (%)	4 (10)
Fracture (%)	1 (3)

**Table 2:** Treatment information.

Characteristic	Statistic *n* = 40
Mean initial positioning time (anesthesia to incision) (mins.) (range)	32 (18–57)
Mean anterior procedure time (incision to anterior close/completion) (mins) (range)	57 (24–145)
Mean repositioning time (for second procedures) (mins) (range)	27 (0–82)*
Mean second procedure time (incision/fixation start to final close) (mins) (range)	73 (6–205)*
Mean total procedure time (anterior and fixation) (mins) (range)	122 (49–274)
Mean total operating room time (mins) (range)	178 (49–342)
Mean anterior procedure estimated blood loss (EBL) (cc) (range)	47 (10–110)
Total number of levels treated	68
Mean number of levels per patient (range)	1.7 (1–3)
Levels treated	*n* (% of levels)
L1-L2	3 (4)
L2-L3	11 (16)
L3-L4	18 (26)
L4-L5	29 (43)
L5–S1 (TLIF)	7 (10)
Supplemental internal fixation (%)	40 (100)
Lateral plating (%)	11 (38)
Bilateral pedicle screws (%)	25 (63)
Direct decompression	
Yes (%)	13 (33)
No (%)	27 (67)
Biologics used	
Osteocel Plus	40 (100)

*In lateral plating following XLIF, repositioning time is zero, as the XLIF exposure is used for placement. Lateral plating also accounts for short second-procedure times in the range. Longer second-procedure times include, in some cases, posterior lumbar interbody fusion at L5–S1 and multilevel posterior fixation, which accounts for the high variability.
